# Double-chambered right ventricle in an adult patient diagnosed by transthoracic echocardiography

**DOI:** 10.1186/1476-7120-5-2

**Published:** 2007-01-04

**Authors:** Minna MD Romano, Rogério G Furtado, Cinthia GF Dias, Mauro Jurca, Oswaldo C Almeida-Filho, Benedito C Maciel

**Affiliations:** 1 Division of Cardiology, Department of Internal Medicine, University Hospital, Medical School of Ribeirão Preto, University of São Paulo, Ribeirão Preto, SP, Brazil

## Abstract

**Background:**

Double-chambered right ventricle is a rare congenital disease frequently misdiagnosed in the adult patient. An anomalous muscle band divides the right ventricle in two cavities causing variable degree of obstruction. Although echocardiography is considered a useful method for the diagnosis of this pathology in children, it has been recognized the transthoracic scanning limitation in adults.

**Case presentation:**

A 29 year-old patient with double-chambered right ventricle presenting mild exercise intolerance referred for follow up of a known ventricular septal defect in whom a complete diagnosis was obtained based only on transthoracic two dimensional echocardiography without the needing of cardiac catheterization.

**Conclusion:**

Based on non invasive echocardiographic diagnosis, patient was referred to surgical correction, which was completely successful.

## Background

Double-chambered right ventricle (DCRV) is a rare congenital heart disorder in which right ventricle is divided in two chambers (high pressure at proximal and low pressure at distal portion) by an anomalous muscle bundle. Although congenital anatomical substrate for this hemodynamic obstruction is recognized, a progression of this obstruction over time is frequently documented. The actual pathophysiological mechanisms responsible for this obstruction are still unclear. At least in some patients, anatomical proximity between moderator band and pulmonary valve or, also, high pressure and blood flow at the out flow tract may contribute to produce obstruction [1-4].

Most patients with DCRV are diagnosed in childhood or adolescence, before 20 years old. Although patients with DCRV seldom present in adulthood, when that happen, the diagnosis can prove elusive and represent a challenge to the clinician [2]. Quite rarely DCRV is an isolated disorder; even though it can be associated to a different congenital heart disease, perimembranous ventricular septal defect (VSD) is the most common coexisting disorder [1,4]. There is a broad spectrum of clinical presentation, depending on the degree of obstruction. The patients may be asymptomatic or present variable symptoms, including: exertional dyspnea, angina, syncope, or dizziness. The physical examination may reveal a harsh systolic ejection murmur in the lower external border.

Even though echocardiography is considered a quite effective method for diagnosis of DCRV in pediatric patients, it has been recognized that transthoracic scanning is less precise for a definitive diagnosis in adults [4,5]. A complete diagnosis using sole transthoracic echocardiography is reported only in 8.3 (1/12) to 15,6% (5/32) in previous series [4,5].

## Case presentation

A 29 year old man, was referred complaining of mild exertional dyspnea, dizziness and chest pain during the last 3 years. These symptoms remained stable during this time. When he was 15 years old, he had meningitis complicated by a cerebral abscess; at that time, in another institution, a harsh cardiac murmur was heard at the left external border and an echocardiographic examination have shown a four millimeter VSD, right ventricular hypertrophy and increased pulmonary flow. The cerebral abscess was surgically drained and the patient, after recovery, remained asymptomatic for the following 11 y. In the current clinical evaluation, physical examination revealed no cyanosis, heart rate of 80 bpm, arterial blood pressure of 110 × 70 mmHg; apical impulse was normopositioned at the 5^0 ^intercostal space. Cardiac rhythm was regular and there was a harsh intense ejection murmur in the low external border.

**Figure 1:**
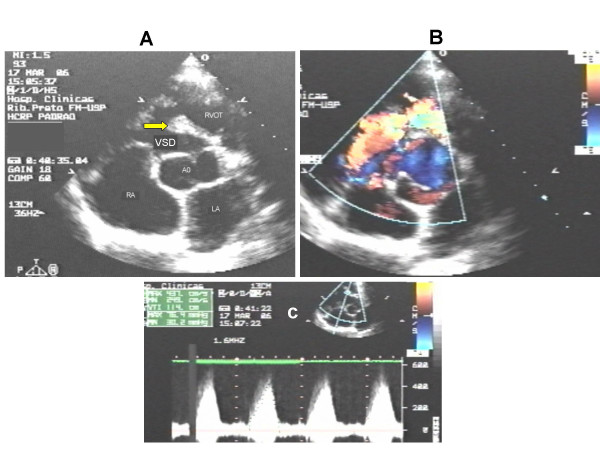
The paraesternal minor axis view shows the large VSD, and a muscular band (arrow) witch shares the right ventricle into two chambers. The right ventricle outflow (RVOF) is free of lesions (panel A). Panel B shows the aliasing phenomena of color at the point of the muscular obstruction, with velocities and gradients represented in panel C.

**Figure 2:**
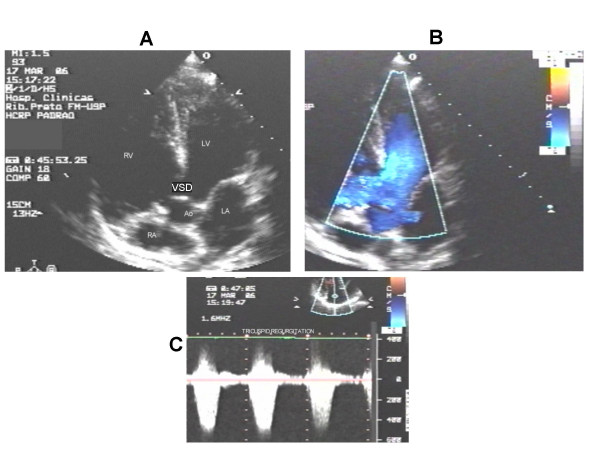
The apical 5 chambers view (panel A) shows the large VSD and right chamber dilatation. The velocity of flow trough the VSD is low (panel B). The RV sistolic pressure elevation is detected trough the tricuspid regurgitation velocities (panel C). RV = right ventricle, RA = right atrium, LV = left ventricle, LA = left atrium

The ECG showed: sinus rhythm, right axis deviation, right ventricular overload and a minor degree of right bundle branch block. Transthoracic echocardiography (Figure 1 and Figure 2) showed: a 17 mm perimembranous VSD associated to a slow velocity flow from left to right ventricle; aortic root was slightly deviated to the right. In addition, there was RV hypertrophy and a muscular septation inside this cavity causing obstruction with a peak gradient of 80 mmHg. There was no obstruction to flow in the right outflow tract and the site of the right obstruction was displaced proximally preserving the right ventricular infundibulum. The pulmonary valve was completely normal.

**Figure 3:**
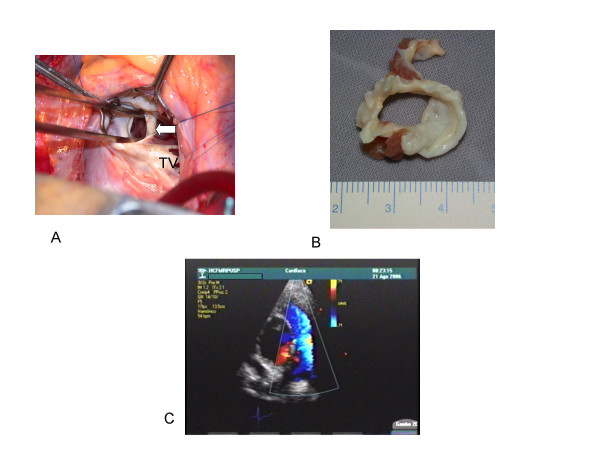
Intraoperatory findings (A and B): A- The transtricuspidic approach reveals the muscular obstructive band (arrow). B- The muscular band resected seemed an obstructive ring. TV = tricuspid valve. Post operatory findings: C- Paraesternal short axis view showing absence of right intraventricular obstruction as documented by color flow mapping.

Based solely on the echocardiographic findings, the patient was referred to surgery which confirmed anatomical and functional abnormalities. VSD was closed and the right anomalous muscle band (Figure 3) was successfully removed. After the procedure, patient remains free of symptoms and medications.

## Conclusion

In patients with DCRV, anomalous muscle band may be located either high, adjacent to the pulmonary valve, or low, near the apex [5]. Even though important limitations for diagnosing DCRV in adult patients by transthoracic echocardiography has been pointed out [4,5], in this patient, a complete anatomical and functional evaluation was provided exclusively by the transthoracic approach.

Considering that this patient had performed an echocardiographic evaluation 15 years ago, when he was diagnosed as presenting VSD and no right obstruction, current data suggest that a progressive obstruction of the right ventricular outflow tract developed during this period, probably influenced by the hemodynamic effects of the VSD. Although the exact mechanism for progression of ventricular obstruction remains unclear [2,3], it has been considered that flow abnormalities related to VSD associated to a congenital underlying anatomical substrate are important determinants for this progressive obstruction [6,7].

Another important aspect is related to the reported difficulty of transthoracic echocardiography for completely establish a definitive diagnosis of DCRV in adults as compared to transesophageal echocardiography and cardiac catheterization [4,5]. The proximity of right ventricular outflow tract from the transducer on precordial approach is accountable for the reported problems. Despite the recognized limitations of this approach, it was possible in this patient to obtain a complete anatomical and functional evaluation. Considering these limitations, it is important, when an increased tricuspid regurgitant gradient, as documented by continous-wave Doppler, is observed in adult patients with congenital heart disease, to consider DCRV as a differential diagnosis for pulmonary hypertension.

## Abbreviations

DCRV = double chambered right ventricle; VSD = ventricular septal defect; RV = right ventricle; ECG = electrocardiogram.

## Competing interests

All the authors declare that they have no competing interests. All authors read and approved the final manuscript.

## Authors' contributions

MMDR conceived the study. selected the images and drafted the manuscript; RGF realized the echocardiographic examinations; CGFD collected the clinical information; MJ participated in the design of the study; OAF reviewed the image selection and participated in the design; BCM was responsible for the coordination of this study and helped to draft the manuscript.
